# The Dark Side of Antibiotics: Adverse Effects on the Infant Immune Defense Against Infection

**DOI:** 10.3389/fped.2020.544460

**Published:** 2020-10-15

**Authors:** Sudhanshu Shekhar, Fernanda Cristina Petersen

**Affiliations:** Institute of Oral Biology, University of Oslo, Oslo, Norway

**Keywords:** antibiotics, infants, microbiota, immunity, infection

## Abstract

Although antibiotics confer significant health benefits in treating or preventing bacterial infections, an accumulating wealth of evidence illustrates their detrimental effect on host-microbiota homeostasis, posing a serious menace to the global public health. In recent years, it is becoming evident that infants, who are subjected to frequent antibiotic exposures due to their vulnerability to infection, reflect increased susceptibility to a wide spectrum of diseases, including infection, in later life. Antibiotics induce perturbations of the microbiota or dysbiosis, which in turn alters the host immune responses against pathogens. In comparison with adults, antibiotic treatments in infants have disproportionate consequences because the infant microbiota represents an evolving system that is unstable and immature until 2–3 years of age. However, relatively less knowledge is available on how antibiotics affect the infant microbiota and immunity. In this review article, we focus on how antibiotic treatment regimens influence the infant innate and adaptive immunity to pathogens in humans and animal models, and make the host susceptible to infections in later life. There is a critical need to better understand the effect of antibiotics on infant immune function, which may have implications for developing effective prophylactics and therapeutics against diseases in infants and adults.

## Introduction

Antibiotics are a boon to human civilization for saving millions of human and animal lives and alleviating infection-inflicted miseries (1, 2). Despite their undisputable health benefits, antibiotics exert unintended collateral effects, including microbiota perturbation or dysbiosis and impaired host immunity to infections and vaccines in humans and mice (3–7). In infants, since the microbiota is immature and the immune system is not fully functional, the consequences inflicted due to antibiotic treatment are more severe and long-lasting than in adults (8–10). Despite these differences in the microbiota and immune landscape, there is a paucity of information on the role and mechanism of antibiotics in regulating immune phenotype and function in infants. Human infants, especially underweight and preterm, are vulnerable to bacterial infections, which cause significant mortality and morbidity worldwide, particularly in low- and lower-middle income countries (11–13). Therefore, infants are subjected to prophylactic and/or therapeutic antibiotic treatments against bacterial infections (14–18). In fact, empiric antibiotic use is a common practice in neonatal intensive care units. Additionally, prepartum antibiotic treatment in mothers is not seldom recommended to reduce the risk of postpartum infections in newborns (19). Around 40% of pregnant mothers and infants receive antibiotics for the prevention and control of infections around the globe (20, 21). Unfortunately, inadequate and inappropriate use of antibiotics in neonates still exists, particularly in developing countries (22, 23). Emerging evidence underscores that antibiotic exposures exert detrimental effects on the infant microbiota and immune system, which render them susceptible to several diseases, including infections, in the times to come (17, 18). This unmasks the dark side of antibiotics, thus warranting in-depth and timely attention of physicians, scientists, and policy makers.

In this review article, we discuss recent findings that stem from human and animal studies focusing on the impact of antibiotics on the infant innate and adaptive immune responses to infections. We also focus on how early life exposure to antibiotics can make the infants prone to infection in later life. A better understanding of the antibiotic effects on the infant immune defense is crucial for developing a rational strategy for disease control and management in infants and adults.

## Microbiota Dysbiosis

The human microbiota consists of commensal microbes, such as bacteria, fungi, and viruses. Out of these, bacterial microbes are the most widely studied and include the dominant phyla – Firmicutes, Bacteroidetes, Actinobacteria, Proteobacteria, Fusobacteria, and Verrucomicrobia (24, 25). They exert a profound impact on the phenotype and function of our body by playing a critical role in the synthesis and absorption of various nutrients and metabolites (e.g., vitamins and short-chain fatty acids), promoting immunity (e.g., pathogen defense), and regulating the brain function and behavior (26, 27). Although still controversial, it is possible that infants are exposed to the maternal microbiota and its metabolites that cross the placenta and enter the amniotic fluid already in the fetal stage (28). However, more recent human and animal studies that have taken in consideration the contamination of laboratory reagents, equipment, and tissue samples with bacterial DNA suggest that fetal and placental tissues are sterile (29–33). Around the time of child labor and birth, a major intergenerational microbiota transfer and colonization occurs, and continues up to 3 years of age (28). This contributes to the development and education of the neonates' immune system, often termed as immunological imprinting, which prepares them to optimally respond to “danger signals” in later phases of life (9). Compositional and functional perturbations of the microbiota, which are possible and usually termed as dysbiosis, can have disproportionate consequences at this stage. With the help of remarkable advances in microbiological techniques, such as metagenomics, and systems biology, it is easier to characterize dysbiosis in terms of alterations in microbial composition, diversity, and function (34). The important factors causing dysbiosis include, but are not limited to, dietary habits, stress, mode of child delivery (cesarean vs. vaginal) and antibiotics (35–37). Antibiotic-driven dysbiosis in particular stands as a major clinically relevant phenomenon that correlates with a myriad number of diseases. It generally leads to decreased microbial diversity in both children and adults, although the nature of the effects depends upon multiple factors, such as antibiotic types and doses (38, 39). The effects of antibiotics on the microbiota can last long after infancy and have long-term consequences for health and disease (40).

## Impact on Host Defense Against Infection

Antibiotics have attracted much attention as one of the most important factors that causes microbial dysbiosis during infancy and are linked to a higher risk of diseases with immune involvement in later life, including infectious diseases (10, 41–44). In case control retrospective studies, prolonged exposure to antibiotic therapy was found to be associated with an increased risk of necrotizing enterocolitis, late-onset sepsis, or death among very low birth weight infants (41, 45–47). By analyzing the stool microbiota and metabolites of preterm infants with seven days of empirical antibiotic treatment, Zhu et al. showed a significant reduction in bacterial diversity and enrichment of pathogens, such as *Streptococcus* and *Pseudomonas* (48). A Danish cohort study demonstrated that maternal antibiotics prescribed before or during pregnancy is associated with an increased risk of infant infection-related hospitalization (49). Epidemiological studies further shed light on how antibiotic use predisposes human infants to susceptibility to diarrhea and respiratory tract infections (42, 43). Although these studies provide crucial indications on increased susceptibility to infection, more convincing and direct evidence originates from studies involving animal models.

Using a mouse model of perinatal antibiotic exposure, Gonzalez-Perez et al. studied the role of antibiotics in causing dysbiosis and regulating immune defense against a systemic vaccinia virus infection (50). Antibiotic treatment of dams led to a significant alteration in the composition of the infant gut microbiota with the predominance of *Enterococcus faecalis* (50). Furthermore, infant mice born from antibiotic-treated, but not sham-treated, dams succumbed to death following intraperitoneal injection with a higher vaccinia virus inoculum, suggesting that antibiotic-driven dysbiosis reduces resistance to infection (50). Even at the lower viral inoculum, control mice exhibited decreased viral titers compared with antibiotic-exposed mice (50). Interestingly, under hygienic environmental conditions, infant mice, which were not exposed to antibiotics, revealed increased susceptibility to vaccinia virus infection (50). This underscores the relationship between the hygiene conditions under which infants are raised and the development of immunity against infection. Recent studies have also assessed the antibiotic-mediated effects on host susceptibility to infections with Gram-positive and Gram-negative bacteria. In a mouse model of bacterial infection, nursing dams were treated with antibiotics in drinking water, and their infants were then exposed via mother's milk (51). The exposed infants, compared with naïve controls, when challenged with *Citrobacter rodentium* 80 days later, showed enhanced pathology (colitis and fecal occult blood), body weight loss, and bacterial burden, which indicates the adverse effects of early life antibiotic usage on pathogen defense in later life (51). Furthermore, the transfer of the antibiotic-perturbed microbiota to germ-free mice led to worsened disease pathologies, suggesting that antibiotic-driven microbiota dysbiosis was responsible for the development of disease phenotype (51). Similarly, antibiotic exposure disturbed the microbial homeostasis in the gut of newborn mice and increased susceptibility to *Streptococcus pneumoniae* infection (52). In addition, neonatal mice exposed to a cocktail of antibiotics demonstrated reduced survivability against challenge infection with *Escherichia coli* serotype K1 or *Klebsiella pneumoniae* compared to control mice (53). Overall, these studies suggest that antibiotics significantly perturb the microbiota composition that leads to long-lasting consequences on host susceptibility to viral and bacterial infections. This highlights the crucial roles played by the commensal microbiota in promoting the infant's resistance to infection (54).

## Animal Models Exploring Antibioitc-Mediated Infant Immunity

Animal models are particularly relevant to establish cause relationships that cannot be directly tested in humans in controlled settings. Although there are limitations in extrapolating findings from animal models, designing experiments that closely resemble the use of antibiotics in humans, including antibiotic combinations, doses, and route of administration, would have the potential to offer more relevant information. For instance, the common practice of extrapolation of antibiotic dose from humans to mice, which is based on the body weight (mg/kg) alone, does not stand appropriate due to physiological and biochemical differences between these two animal species that influence pharmacodynamics and pharmacokinetics of antibiotics. Hence, better approaches, such as allometric scaling based on normalization of dose to body surface area, are needed to calibrate the drug dose (55). While mice are the preferred animal models for antibiotic-related studies in neonates, the use of large animals like pigs may provide a better alternative due to (1) anatomical and physiological similarities with humans, and (2) large-sized neonates allow easier therapeutic manipulations like intramuscular and intravenous injections.

## Modulation of Immune Responses to Pathogens

### Innate Immunity

**Figure 1:**
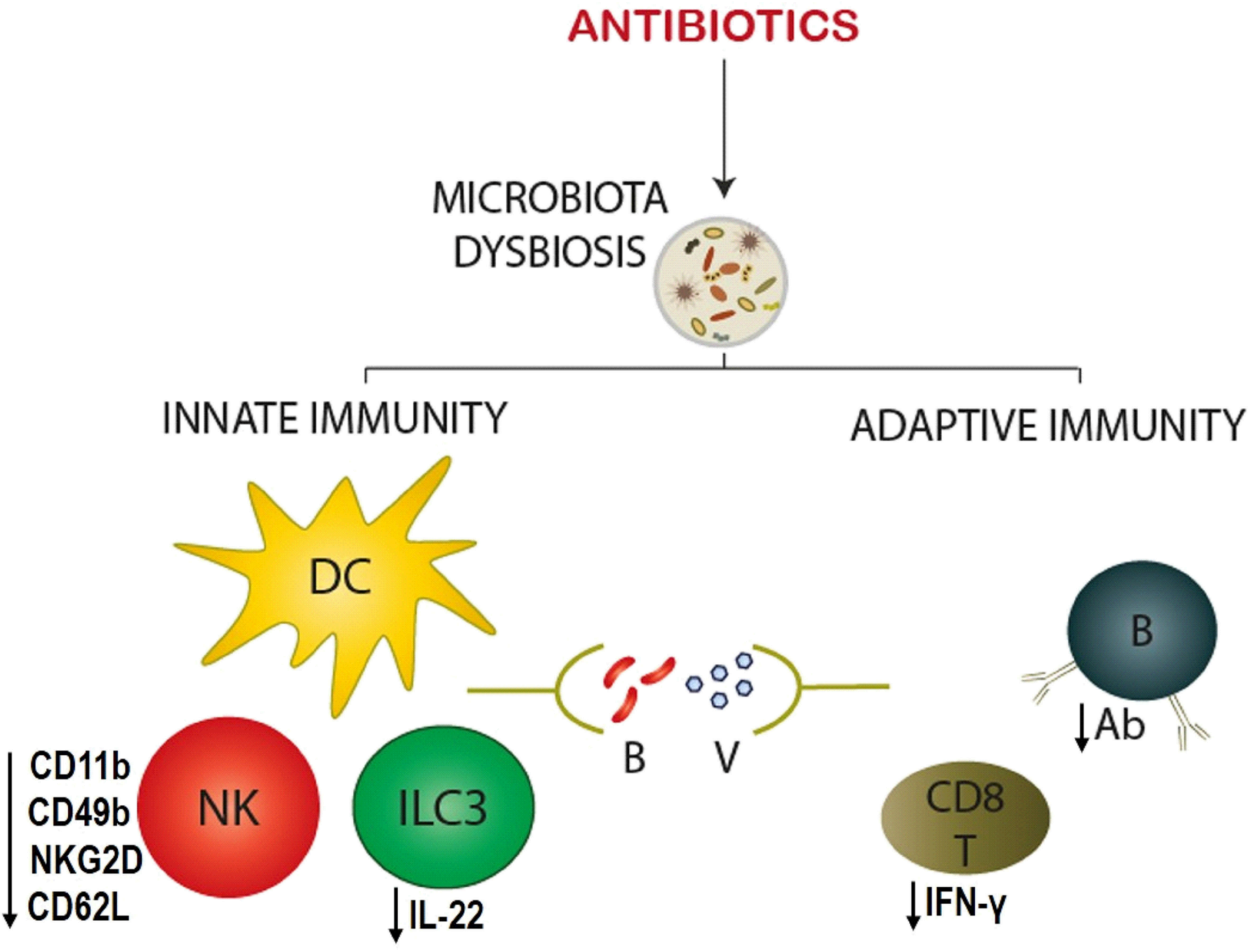
Antibiotic-driven modulation of the infant immune responses against infection. Antibiotic exposure to infants causes the microbiota dysbiosis, which in turn alters innate and adaptive immune responses to bacterial (B) and viral (V) pathogens. Innate immune cells - dendritic cell (DC), natural killer (NK) cell, and type 3 innate lymphoid cell (ILC3) – show significant changes in their phenotype, and function. NK cell exhibits reduced maturation (CD49b) and activation (CD11b, CD62L, and NKG2D) markers, whereas, ILC3 secretes decreased quantities of cytokines (IL-22). On the other hand, adaptive immune cells – B and CD8+ T cells – reveal a deficiency in their effector function by producing decreased antigen-specific antibodies (Ab) and CD8+ T cell responses (IFN-γ), respectively.

Antibiotic-driven dysbiosis imparts profound impact on the generation of the infant innate and adaptive immune responses toward pathogens and vaccines (Figure 1) (50, 56–58). Antibiotic exposures during infancy influence innate immune cells, such as dendritic cells (DCs), natural killer (NK) cells, and innate lymphoid cells (50, 52). DCs are the most potent antigen-presenting cells that act as a connecting link between innate and adaptive immunity. Following infection with vaccinia virus, infant mice born out of antibiotic-treated dams had a reduced frequency and absolute number of splenic DCs characterized by CD11c^hi^MHC-II^hi^ compared with control mice (50). Although no significant difference was found in the numbers of the major subsets of splenic DCs, CD8α+ and CD8α-, antibiotic exposed mice had a higher frequency of CD11b-CD103+ DCs (50). However, the function of DC and its subsets was not explored in this study. Considering the fact that CD8α+ and CD11b-CD103+ DCs, often referred to as type 1 DCs, play a pivotal role in cross-presentation of exogenous antigens to CD8+ T cells to confer antiviral immunity, it would be interesting to perform a detailed analysis of the phenotype and function of these DC subsets in relation to viral infections (59). Of note, antibiotic treatment has also been reported to reduce the expression of IFN-γ responsive genes in peripheral blood monocytes and DCs due to epigenetic changes (60). Similar to changes in splenic DCs after vaccinia virus infection, NK cells in antibiotic-exposed infants exhibited remarkable alterations in terms of frequency and phenotype (50). There was a decreased influx of these NK cells following infection, and reduced expression of activation markers, CD11b, CD62L, and NKG2D, compared to controls (50). Furthermore, mouse model shows that antibiotic treatment depletes microbiota, reduces circulating peptidoglycan and leads to impaired neutrophil-mediated killing of *Streptococcus pneumoniae* and *Staphylococcus aureus* (61). Thus, these data indicate that hindrance in microbial colonization by antibiotics during early life could contribute to inefficiency of innate immune cells in generating optimal immunity.

Group 3 innate lymphoid cells (ILC3) that secrete IL-22 (IL-22+ILC3), a cytokine critical in lung epithelial repair and pathogen defense, have been shown to play an important role in infant immunity against *S. pneumoniae* infection (52). IL-22+ILC3 from antibiotic-exposed newborn mice had decreased ability to preferentially migrate to the lungs, but not the spleen or intestine, compared to these cells isolated from control mice. And, the decreased migration was associated with lower expression of chemokine receptor (CCR) 4, which is crucial for homeostatic trafficking of T cells to the lungs (52). However, reversing the antibiotic-mediated dysbiosis by the microbiota reconstitution restored the migratory abilities of IL-22+ILC3 (52). Using a co-culture system of intestinal CD103+CD11b+ DCs from the antibiotic-exposed or -unexposed infants with the lung ILC3, it was further shown that CD103+CD11b+ DCs from antibiotic-exposed infants were less efficient than CD103+CD11b+ DCs from unexposed mice at inducing CCR4 expression on IL-22+ILC3 (52). In contrast, CD103-CD11b+ DCs failed to induce such effect, suggesting differential functional abilities of these DC subsets. These data shed light on how early-life antibiotic exposures adversely affect the migratory pattern of the lung ILC3, which contribute to protective immunity to pneumococcal lung infection.

### Adaptive Immunity

Lynn et al. performed an in-depth study on how early-life antibiotic-driven dysbiosis influences vaccine immune responses in mice (57). The mouse infants exposed to antibiotics were first immunized with multiple clinically relevant vaccines - BCG, MenB/MenC, PCV 13, or Hexa - which are used for protection against tuberculosis, meningitis, pneumococcal disease, and six different diseases (diphtheria, tetanus, pertussis, hepatitis B, *Haemophilus influenzae* type b infection, and polio), respectively. Up to 12 weeks after immunization, these mice had significantly impaired antigen-specific IgG antibody responses to vaccines compared to sham-treated mice (57). Although booster doses of the vaccines to some extent raised the antibody titer specific to antigens, the effect was transient and unsustainable. Since no difference was noticed between the total IgG antibodies of antibiotic- and sham-treated mice, it is likely that impaired antibody responses that are specific to vaccinations did not arise due to a major defect in B cell function (57). Moreover, subcutaneous immunization of antibiotic-exposed murine infants (7 days old) with ovalbumin and complete Freund's adjuvant resulted in reduced antigen-specific serum antibody titers (58). Thus, antibiotic exposure in early-life impairs humoral responses that are crucial for protective immunity to the majority of vaccines.

Antibiotic treatment of infant mice also alters T cell immunity to infections (50, 62–64). Upon infection with vaccinia virus, the functional profile of splenic T cells from the mice exposed to antibiotics was different from control mice (50). Flow cytometric analysis showed that the absolute numbers of CD8+ T cells that express IFN-γ were significantly lower in antibiotic-treated mice. Similar findings were observed following *in vitro* and *ex-vivo* stimulation of CD8+ T cells with T cell receptor (TCR) and CD28 agonists. Interestingly, these CD8+ T cells showed down-regulation of anti-apoptotic (Bcl-2) and up-regulation of exhaustion (PD-1) markers, implying that antibiotic-mediated dysbiosis could cause intrinsic defects in T cell effector function (50). It was further shown that CD8+ T cells from infant mice born to antibiotic-treated dams exhibit alterations in T cell receptor signaling, and that TLR4 stimulation with LPS *in vivo* and *in vitro* partially restores IFN-γ production and reduces mortality against vaccinia virus infection (62). Likewise, treatment of mice with antibiotics for 4 weeks resulted in a reduction in the function of antigen-specific CD4+ and CD8+ T cells, and this immune impairment was rescued by distal or local injections of TLR ligands (65). But how does dysbiosis alter the function of T cells remains poorly understood. It could be that dysbiosis leads to altered production of metabolites, such as short-chain fatty acids, by commensal bacteria, which play a key role in the generation of regulatory T cells (63). In contrast to impaired antigen-specific T cell responses (50), CD4+ T cells isolated from antibiotic-treated mice, which were immunized with PCV 13, produced higher levels of cytokines, such as IFN-γ, than the cells isolated from sham-treated mice (57). Similarly, increased IFN-γ production was found when splenocytes isolated from antibiotic-exposed mice receiving immunizations with Hexa were stimulated (57). Upon *in vitro* polyclonal stimulation with phytohaemagglutinin, peripheral blood mononuclear cells from antibiotic-exposed piglets secreted enhanced levels of IFN-γ compared to unexposed mice (64). Furthermore, following heat killed *Salmonella* Typhimurium challenge, peripheral blood gene expression of IFN-γ, IL-6, and IL-2 was significantly upregulated in antibiotic-exposed piglets (64). The discrepancy in these findings could be attributed to different animal models, antibiotic combinations, experimental design, and infection/vaccine challenges. Overall, these data have important implications for child vaccination programs due to the fact that human infants are widely exposed to antibiotics worldwide.

Relatively fewer studies have attempted to explore the effect of antibiotics/dysbiosis on immune cells in human infants (66, 67). Oosterloo et al. studied the effect of neonatal antibiotic treatment on the expression of 84 different markers on circulating immune cells in a subgroup of term infants from the INCA-study at 12 months of life and evaluated whether the immune profile was associated with allergic disorders (eczema and wheezing) and infantile colics (67). They found a significant association of broad-spectrum antibiotic use with immune inflammatory markers (e.g. sVCAM-1, sCD14, sCD19, sCD27, IL-1RII, sVEGF-R1, and HSP70) at 1 year of age, and identified immune profiles for eczema, wheezing and colics some of which were independent of antibiotic treatment (67). In another study, infants showing gut microbiome dysbiosis at 12 weeks of life dominated by Bacilli and Gammaproteobacteria had more activated T cell populations - CD4+ TEMRA, CD4+ TEM, and CD8+ TEMRA cells - compared with normal microbiome infants (66). These studies highlight the importance of the interaction between the commensal microbiota and host for the development of immune system.

## Conclusions and Future Directions

Antibiotics are often unavoidable drug of neonatal clinical care to prevent and treat bacterial infections that inflict considerable socioeconomic burden on society. This is mainly due to challenges posed in accurate diagnosis of neonatal sepsis. There is an increasing awareness about serious consequences of antibiotic use in infants. Antibiotics promote changes in microbial ecology, which have been implicated in altered immune responses against pathogens and vaccines, and increased susceptibility to infections in later life. Understanding how antibiotic treatments during infancy shape the microbiota and immunity is crucial for designing better prophylactic and therapeutic strategies. It remains unclear whether antibiotics can directly modulate the infant immune function. Recent studies involving germ-free adult mice have provided interesting findings that demonstrate how antibiotics can regulate host immunity, including pathogen defense, in a microbiota-independent fashion (68, 69). But these findings await confirmation in infants. Future studies need to address the following questions: 1. What are the underlying mechanisms by which antibiotic-driven dysbiosis in infants control the immune responses to vaccines and infections? 2. Do antibiotics directly act on the infant immune system without involvement of the microbiota? 3. How can the negative effects of antibiotic use in infants be neutralized in order to strengthen antibiotic stewardship programs? 4. How to develop better animal models to study the antibiotic effect on the microbiota and immunity?

## Author Contributions

SS and FP wrote the manuscript, assisted in the conception of the manuscript, and gave approval of the last version to be published.

## Conflict of Interest

The authors declare that the research was conducted in the absence of any commercial or financial relationships that could be construed as a potential conflict of interest.
